# Regulation challenge of tissue engineering and regenerative medicine in China

**DOI:** 10.4103/2321-3868.118927

**Published:** 2013-09-18

**Authors:** Liang Chen, Chunren Wang, Tingfei Xi

**Affiliations:** 1 Center for Biomaterials and Tissue Engineering, National Institutes for Food and Drug Control, Beijing, China; 2 Center for Biomedical Materials and Tissue Engineering, Peking University, Beijing, China; 3 Center for Biomedical Materials and Tissue Engineering, Peking University, Beijing, Room 402, Chengfu Lu 205, District of Haidian, China

**Keywords:** Regenerative medicine, tissue engineering, medical product, regulation, techniquep

## Abstract

The current regulatory status in the USA, European Union (EU), Japan, and China, associated with the clinical application of tissue engineering and regenerative medicine is presented. It is found that similar regulatory framework has been constructed in the USA and EU, in which risk-based regulatory strategy is used to determine which regulatory mode is more desirable between medical technique and medical product. Also, it is suggested that two-tier regulatory framework of medical products be constructed, with the first level built on existing and newly introduced regulatory provisions and the second technical level encompassing all the technical requirements. In China, the demarcation line between medical technique mode and medical product mode is not made clear and coherent and flexible regulatory framework has not been intentionally designed. If the recommendations concerning the desirability of regulatory framework will be adopted in China, it means the present application of medical technique mode should be reconsidered and adjusted based on the risk analysis. Furthermore, the construction of two-tier regulatory framework which is tailored to meet the demands of development of medical products of tissue engineering and regenerative medicine still remains a challenge.

## Introduction

For patients with extensive burns, the restoration of skin barrier function could make the difference between life and death, and it was this acute need that drove the initiation of tissue engineering in the 1980s.[1] With the reliable keratinocyte culture technique, the epithelial sheets [known as cultured epithelial autografts (CEA)] produced from a small skin sample have been used to handle burn wounds.[2] Although the split-thickness skin autografts are the “gold standard” for burn wound closure, it is limited by massive tissue destruction and/or unsuited donor sites (e.g. hands and face). A number of approaches with cultured skin cells have been developed to restart the wound healing of burns and chronic wounds. The allogeneic neonatal fibroblasts and keratinocytes are designed to be delivered in a fibrin spray, and this has shown preliminarily an excellent result in the clinical trial of chronic venous leg ulcer treatment.[3] DERMAGRAFT®, composed of fibroblasts, extracellular matrix, and a bioabsorbable polyglactin mesh scaffold, has been approved as a dermal substitute used for the treatment of full-thickness diabetic foot ulcer.[4] These products and techniques are so different from each other, as they might be based on either the use of acellular matrices, cultured cells, or a combination of both. But essentially, they can be thought of as sprouting from tissue engineering and regenerative medicine, which aim at the repair and regeneration of the structure and function of damaged tissue and organ.

**Table:** 

Access this article online	
**Quick Response Code:**	**Website:** www.burnstrauma.com
	**DOI:** 10.4103/2321-3868.118927

Besides the skin, the researches in tissue engineering and regenerative medicine are involved with almost every kind of human tissue and organ. There are more and more evidences showing that the related research results might be promising in the clinic as an alternative to traditional clinic treatment. But on the other side, the medical techniques and medical products derived from tissue engineering and regenerative medicine can be very complex, which are different from ordinary medicinal products or medical devices. It is unlikely that all aspects of tissue engineering and regenerative medicine can be encompassed by current legislation, for example, in relation to medicinal products, or medical devices, or clinical trials.[5] New regulatory framework should be developed to oversee the introduction of these advanced medical products and techniques into clinic.

## Concepts of tissue engineering and regenerative medicine

To establish the regulatory framework, the first step might be the development of the definitions of tissue engineering and regenerative medicine, which define the boundaries of the area that will be regulated in this way. In the fourth annual National Institutes of Health Bioengineering Consortium (BECON) Symposium held in 2001, the regenerative medicine was equally referred to as reparative medicine or tissue engineering, for which the definition presented was that the regeneration and remodeling of tissue *in vivo* for the purpose of repairing, replacing, maintaining, or enhancing organ function, and the engineering and growing of functional tissue substitutes *in vitro* for implantation *in vivo* as a biological substitute for damaged or diseased tissues and organs.[6] In fact, the term of tissue engineering was proposed early than that of regenerative medicine. In this area, cells are typically combined with matrices to achieve new tissue formation.[7] With the new field of stem cell biology emerging, it is found that the stem cell holds great promise in its potential for therapy using tissue engineering strategies. Thus, the term regenerative medicine is recently more referred to, which aptly describes the intersection of stem cell science with tissue engineering.[8] It involves typically the systemic delivery of stem cells in the hope of reversing degenerative disease or stimulating repair post injury.[9]

Furthermore, the term regenerative medicine combines the elements of tissue engineering and stem cell science to encompass the broad range of scientific disciplines that are necessary to further the field.[10] The approaches under development have evolved from the earlier focus on replacement to the recent inclusion of repair and regeneration. Replacement involves the growing or fabricating of tissues and organs outside the body and then implanting them. Repair is the biological effect at the cell and molecular level, including the repair of DNA. Finally, regeneration is to literally grow *in vivo* a new tissue or organ.[11] It might also be noted that whether one’s strategy is replacement, repair, or regeneration, the approaches one uses can involve cells alone, cells seeded in a scaffold, or even a scaffold alone.[11] But the formation of tissue and organ is achieved by cells, whether the cells are intentionally introduced *in vitro* or mobilized *in vivo*. Cells sense signals from soluble molecules, from the substrate/matrix to which the cells are adherent, and from the mechanical or physical forces acting on them. So, it is usually thought that cells should be the core element of medical products and techniques derived from tissue engineering and regenerative medicine.

## Regulation mode in clinic application: Medical technique or medical product

For the introduction of tissue engineering and regenerative medicine into clinical practice, specific regulatory framework is required to be designed. First of all, it should be considered that when tissue engineering and regenerative medicine are applied in clinic, they ought to be adopted as medical techniques or as more regulated medical products, which represent very different regulatory requirements.

In the USA, if the cells are minimally manipulated and homologously used only, are not combined with a drug or device, and are not for metabolic use when from an unrelated donor [Table 1], according to the Food and Drug Administration (FDA), the usage of cells can be carried out as a clinic technique in a well-equipped hospital without FDA approval,[12,13] in which only control of communicable diseases is required in the procedure involving cells. For instance, autologous bone marrow — derived cells can be harvested from peripheral blood following stimulation with appropriate growth factors, definite cell populations are isolated by specific surface markers, and the concentrated cell preparation can be re-infused in the systemic circulation or in the affected organ.

But if the manipulation and usage of cells are beyond the above criteria, for example, the culture, expansion, and/or differentiation of the cells, or cells combined with biomaterial scaffold, these processes are more elaborated and require more complex and expensive facilities, and more risks should be taken care of. When tissue engineering and regenerative medicine are translated into clinical application in such matters, there should be manufacture quality control and they should be regulated by the FDA as medical products.

**Table 1:** Current status of regulation of cell-based tissue engineering and regenerative medicine

Country/region	Current status
USA	If it is intended to be used as medical technique in clinic, the following conditions must be fulfilled: Cells are (1) minimally manipulated, (2) homologously used, (3) not combined with a drug or device, and (4) have no systemic effect and do not depend upon the metabolic activity of living cells for the primary function (except for autologous use or allogeneic use in a first-degree or second-degree blood relative). Otherwise, it will be regulated as a medical product with premarket approval requirement
EU	The following conditions need to be fulfilled: Cells (1) are not subjected to substantial manipulation, (2) have the same function, and (3) are not combined with medical devices, or if cells are prepared and used as a custom-made product for an individual patient in a hospital, their usage might be treated as medical technique. Otherwise, they should be presented as a medicinal product
Japan	It is permitted to carry out human stem cell clinical research, even though stem cells are substantially manipulated or combined with non-cellular components
China	There have not been any criteria related to the manipulation and usage of cells, which can be used to determine whether the medical technique mode or the medical product mode is desired

In the European Union (EU), similar strategy is adopted to determine the regulation mode of tissue engineering and regenerative medicine in clinic. If cells are only subjected to separation, concentration, purification, filtering, cryopreservation, or vitrification, and have the same function and are not combined with medical devices, their usages for the intended regeneration, repair, or replacement of tissue and organ might be treated as medical techniques [Table 1]. Otherwise, they should be presented as medicinal products which are regulated for the marketing authorization at the European community level.[14]

In Japan, if the use of stem cell is aimed at the regeneration of organs or tissues lost or damaged due to injury or disease, it is permitted to carry out human stem cell clinical research, even though the processing of cells is beyond the minimal manipulation, such as artificially induced proliferation, drug treatments intended to activate cells or elicit other effects, modification of biological properties, combination with non-cellular components, etc.[15] This is deemed a pragmatic regulatory strategy since there is no proper “regulatory approval pathway” for the commercialization as medical products, for instance, some important rules have been lacking for preclinical studies and clinical assessment.[16] This has hampered the development of application in clinic as medical products of tissue engineering and regenerative medicine.

In China, there seem to have two parallel regulatory regimes that can both be applied to the transformation to the clinic of tissue engineering and regenerative medicine. They are not mutually exclusive regulations. Medical technique or medical product, each of them can be the choice, no matter whether the cells are minimally or substantially manipulated.[17,18] The current regulatory situation might reflect the present status of research and development of tissue engineering and regenerative medicine in China, which are mainly in the professional medical institutions where the clinicians try to carry out some tissue engineering and regenerative medicine based treatments. But on the other side, it also reflects, to some degree, the predicament in the way of developing medical products.

The above information represents, to some degree, the current regulatory situation of tissue engineering and regenerative medicine [Table 1]. It is obvious that various kinds of framework are designed by regulators. Just based on the mentioned cell-related criteria, the demarcation line is made clear by regulators of the USA and EU to determine which regulatory mode is more desirable between medical technique and medical product. This regulatory scheme represents an ambitious attempt to set up a coherent and flexible framework for tissue engineering and regenerative medicine. In most researches, the cells might be subjected to substantial manipulation, or nonhomologous use, or combination with drugs or medical devices, or have systemic function/ metabolic function. In such circumstances, the risks such as those related to transmission of communicable disease, control of processing, and clinical safety and effectiveness are significant, in which the desirability of regulation mode of medical technique is challenged in nature. The establishment of medical product mode should be the core of regulatory framework for the introduction of tissue engineering and regenerative medicine into clinic.

## Current regulation as medical product in the USA and EU

When medical products derived from tissue engineering and regenerative medicine are applied in clinic, these products are intended to have the potential of repair, replacement, or regeneration of damaged tissues and organs. These are generally considered as the typical characteristics that might be used to distinguish these medical products from the others, since these therapeutic effects are dependent on the biological function of cells, which are different from the effects produced by the electromechanical devices or prosthetic implants. But a unanimously accepted concept of medical products derived from tissue engineering and regenerative medicine is yet to develop. Several terms have been proposed, for example, “tissue engineered medical product” (TEMP) and “tissue engineered product” (TEP), and several definitions have been given which cover the characteristics of these medical products.

The term TEMP has been defined in a standard document of the American Society for Testing and Materials (ASTM). TEMP is defined as “a medical product that repairs, modifies, or regenerates the recipient’s cells, tissues, and organs or their structure and function, or both.”[19] In the USA, this terminology standard has been included in the FDA-recognized consensus standards database. In this definition, what comprises TEMPs is not clarified, and just in the following sentences is given the explanation as “TEMPs derive their therapeutic potential from various components used alone or in various combinations. The components might be biological products (i.e. cells, organs, tissues, derivatives, and processed biologics), biomaterials (i.e. substrates and scaffolds), biomolecules, devices, and drugs.” This means if a TEMP is only composed of cell, biomaterial scaffold, or chemical alone, it might be correspondingly thought as a biological product, medical device, or drug, respectively, and is included in the regulatory regimes that have been developed for these medical products. But in more complex instances, the TEMPs might be composed of any combinations of drugs, devices, and biological products, where such applications fall under the category of combination products.

The regulatory framework for combination products has been developed in the FDA during the last decade. A combination product is assigned to an agency center or alternative organizational component that will have primary jurisdiction for its premarket review and regulation. The assignment is based on determination of the primary mode of action (PMOA) of the combination product.[20] For example, if the PMOA of a device—biological combination product is attributable to the biological product, the agency responsible for premarket review of that biological product will have primary jurisdiction for the combination product. Apligraf is a bilayered tissue construct consisting of allogeneic keratinocytes, fibroblasts, and bovine type I collagen. At first, Apligraf had been approved as a medical device for the treatment of venous leg and diabetic foot ulcers;[21] but now following the jurisdiction mechanism of the combination product, this product is diverted to a biologics license application (BLA) regulatory pathway for the treatment of surgically created gingival and alveolar mucosal surface defects in adults.[22]

In the EU, the term TEP is proposed and the definition is: “A product that contains or consists of engineered cells or tissues, and is presented as having properties for, or is used in or administered to human beings with a view to regenerating, repairing, or replacing a human tissue.”[14] The definition of TEP is similar to that of TEMP recognized by the FDA, where the tissue repairing, replacing, or regenerating potential of the product is emphasized, but there exist significant differences. The engineered cells are considered as indispensable components of TEPs, and that TEPs are classified as advanced therapy medicinal products might be attributed to this. When the TEP incorporates, as an integral part of the product, one or more medical devices, it is thought as the combined advanced therapy medicinal product because where a product contains viable cells or tissues, the pharmacological, immunological, or metabolic action of those cells or tissues should be considered as the principal mode of action of the product.

When medical products derived from tissue engineering and regenerative medicine are intended to be used in clinic, their components and action modes will be so diversified and the attributes of these medical products might be different. The regulatory strategy has to make a difficult balance between the possibility for patients to gain rapid access to promising products and appropriate guarantees on safety and quality. It is, therefore, suggested to opt for a two-stage regulatory strategy,[23] with the first level built on the existing and newly introduced regulatory provisions and the second technical level encompassing all the technical requirements for the whole development process, from production, handling, storage, and transport to traceability of the donor. Since tissue engineering and regenerative medicine are emerging and fast-moving fields, the technological development is not fully foreseeable. It is worth noting that the capability of the regulation strategy to adapt in a timely manner to scientific progress relies, therefore, on its technical level, which can be updated and revised in a flexible and rapid manner.

As living cells, biomaterials, and growth factors might be involved in tissue engineering and regenerative medicine, the existing provisions in relation to medicinal products and medical devices can be applicable. Cells are the key element in tissue engineering and regenerative medicine. In the USA, to cope with the challenge of cellular and tissue-based products, the “Tissue Action Plan” was initiated in 1998. Until 2005, three rules about human cells, tissues, and cellular and tissue-based products had been issued: “establishment registration and listing,” “eligibility determination for donors,” and “current Good Tissue Practice.” These three rules, combined with the existing rules for drug, biological product, and medical device, have constituted the rounded regulatory regime for medical products derived from tissue engineering and regenerative medicine. At the technical level, many important guidance documents have been also issued timely,[24] for example, “guidance of chemistry, manufacturing, and control (CMC) information for human somatic cell therapy investigational new drug applications.” In the EU, the integrated regulatory framework is on the way. The new regulation No. 1394/2007 is provided for TEP marketing authorization procedure. Some technical guidance documents have been issued, for example, “guideline on cell-based medicinal products.”[25] But some technical requirements, like Good Manufacturing Practice and Good Clinical Practice, have not been developed in detail yet.[23]

## Current status of regulation and challenge in China

In China, if the application of tissue engineering and regenerative medicine is intended to follow the regulation mode of medical product, there are several provisions and guidance that can be referred to. When the product is composed of cell and biomaterial, it is considered as a combination product,[26] which might be applied for the product classification at first, if this kind of combination product has not obtained the marketing approval before in China. This is also based on a determination of the PMOA of the combination product to define it as a drug or a medical device. At present, there has been only one such combination product which has obtained the marketing approval in China, the “Activskin” tissue-engineered skin composed of human keratinocytes, fibroblasts, and collagen, and approved as a medical device.[27] Following the given classification of medical device, other tissue-engineered skin products also might apply for marketing approval as medical device either, in which guidance “Requirements for Tissue Engineered Medical Product Research and Registration Application”[28] should be referred to. But this guidance is limited; it only covers tissue-engineered medical products applying for registration as medical device.

In this guidance, the term “tissue engineered medical product” is proposed and the definition is provided as a medical product that is produced by the principle and technique of tissue engineering, and repairs, modifies, or regenerates the structure and function of tissues and organs. It is worth noting that human somatic cell therapy product consisting of cells only is excluded from the scope of “tissue engineered medical product” even if the human somatic cell therapy product is intended to repair, modify, or regenerate the structure and function of tissues and organs. So, if the products derived from tissue engineering and regenerative medicine consist of cells only, they will fall in the scope of cell therapy products and the document “guidance for human somatic cell therapy research and product quality control” should be followed to carry out the preclinical studies and clinical assessment.[17] The requirements for cell therapy in this guidance can also be applicable to the cell component of tissue engineered medical product as the combination product. Whether the cells are used alone or combined, the biological characteristics of the cells might be substantially altered as a result of their manipulation, so quality control of processing is mandatory. The principles included in the document “guide for processing cells,tissues,and organs” can serve as guidance for developing these manufacturing procedures of tissue engineered medical products.[29]

It is recognized that based on existing legislation in relation to drugs and medical devices, and newly introduced regulatory provisions, a regulatory pathway to premarket application of the products derived from tissue engineering and regenerative medicine has been forming in China. However, there are only few technical guidelines about the manufacture practice, preclinical studies, clinical assessment, etc. This means the two-level regulatory strategy has not been well developed and the regulatory framework of tissue engineering and regenerative medicine not yet finished. Especially when we are aware of the coexistence of medical product mode and medical technique mode in many cases, it is likely that a coherent regulatory framework of tissue engineering and regenerative medicine has not been intentionally designed.

If cells are associated with matrices or scaffolds as the integral, they are regarded as combination products with the need for premarket approval. The matrices or scaffolds which are combined with manipulated cells should meet the requirements of medical device regulation. But in the case of autologous cells that are combined with biomaterial scaffold to repair the structural tissue (skin, bone, cartilage, etc.), it is allowed to be carried out as a clinical medical technique,[30] and the requirements for matrices and scaffolds are few; just the quality standard of biomaterial and the corresponding testing report of an authorized institute should be provided. More evidences of conformity of the medical device regulation, including the requirements for manufacture practice are absent.

Cell transplantation technique has been approved as a third-class medical technique in clinic.[18] Except that stem cells are excluded from transplantation, there are nearly no other limitations to the usage of viable cells, no matter whether they are autogeneic or allogeneic, minimally or substantially manipulated. This means some mature/functionally differentiated cell-based treatments of tissue engineering and regenerative medicine can be used as medical techniques in clinic; but at the same time, they might also fall into the scope of cell therapy products. If the more desirable regulatory mode is not defined, the guarantee on the safety and effectiveness in clinic might be impaired or weakened.

Stem cell is the enabling factor in the repair and regeneration of tissues and organs. The usage of stem cells has not been approved as the third-class medical technique in clinic since the regulation of medical technique was implemented in 2009. But there were once so many stem cell-based treatments offered at a high cost in the absence of rigorous scientific and regulatory requirements.[31] There are serious concerns about the safety and efficacy of such treatments. They might lack pharmacological or toxicological data from non-clinical studies and generally, there are no peer-reviewed publications to demonstrate their efficacy. Until recently, the loose regulation on the usage of stem cells in clinic has been changed in China. The usage of stem cells is restricted to the clinical experimental research. That means it cannot be advertised and there is no charge for the use of stem cells. The application of clinical experimental research of stem cells should be subjected to a centralized authorization procedure by a top competent authority, involving a uniform scientific evaluation of the quality, safety, and efficacy, which should be carried out according to the highest possible standards.[32] However, the regulation on the stem cell clinical experimental research is not applicable to the clinical trial in the development process of stem cell product. For stem cell products, some important guidelines on the preclinical study and clinical assessment are lacking.

It is obvious that the medical technique mode is adopted in so many cases of clinical application of tissue engineering and regenerative medicine. This is deemed a pragmatic regulatory strategy. It tentatively bridges the gap between the very rapid progress made in tissue engineering and regenerative medicine and their application in the clinic. The medical technique mode is not involved with the hazards related to the defective products or unforeseen mechanisms that can affect thousands of patients, but this situation still requires careful analysis in terms of the risk-benefit weighing. The medical technique mode is preferred to be adopted in conditions of less risk, where the cells are minimally manipulated, homologously used only, and are not combined with drugs or medical devices. In the cases where the cells are subjected to substantial manipulation, or are not homologously used, or are combined with medical devices, there will be more risks which should be taken care of, such as those related to transmission of communicable disease, control of processing, and clinical safety and effectiveness concerns. Although the quality and safety related rules are required to be established in accordance with the relevant criteria of medical product, how these are established depends on individual medical institution where the medical technique is carried out. There will not be uniform regulation without a centralized authorization procedure. On the contrary, the regulatory regime of medical product is characterized by the centralized authorization procedure in which the scarcity of expertise in the area can be overcome and a uniform scientific evaluation of the quality, safety, and efficacy can be ensured. So, the necessity of a set of flexible regulatory requirements in the clinical application of tissue engineering and regenerative medicine is evident, but regarding the choice of medical technique mode or medical product mode, it should be decided on the risk-based strategy in which more thorough requirements apgenerative medicine are translated into clinical application in such matters, there should be manufacture quality control and they should be regulated by the FDA as medical products.

## Conclusion

The above information represents the current regulatory situation associated with clinic application of tissue engineering and regenerative medicine, in which it is obvious that similar regulatory frameworks are constructed in the USA and EU The risk-based regulatory strategy is highlighted which should be the determinant of the choice of regulatory mode. The mode of medical technique means there are accepted and limited risks imposed on patients. To cope with the hazards related to the medical products derived from tissue engineering and regenerative medicine and promote the commercialization, the two-tier regulatory framework composed of regulatory provisions and technique guidelines is suggested to be developed. In China, the adoption of risk-based strategy means there should be reconsideration and adjustment of the present application conditions of medical technique mode. On the other hand, the construction of two-tier regulatory framework which is tailored to meet the demands of development of medical products of tissue engineering and regenerative medicine still remains a challenge.
